# Draft Genome Sequence of Lentilactobacillus kosonis NBRC 111893, Isolated from a Japanese Sugar-Vegetable Fermented Beverage called Kôso

**DOI:** 10.1128/mra.00775-22

**Published:** 2022-09-22

**Authors:** Tai-Ying Chiou, Wataru Suda, Kenshiro Oshima, Masahira Hattori, Chiaki Matsuzaki, Kenji Yamamoto, Tomoya Takahashi

**Affiliations:** a Department of Biotechnology and Environmental Chemistry, Kitami Institute of Technology, Kitami, Hokkaido, Japan; b Japan Department of Computational Biology, Graduate School of Frontier Sciences, The University of Tokyo, Kashiwa, Chiba, Japan; c Laboratory for Microbiome Sciences, RIKEN Center for Integrative Medical Sciences, Yokohama, Kanagawa, Japan; d School of Pharmacy, Kitasato University, Sagamihara, Kanagawa, Japan; e Research Institute for Bioresources and Biotechnology, Ishikawa Prefectural University, Nonoichi, Ishikawa, Japan; f Center for Innovative and Joint Research, Wakayama University, Wakayama, Wakayama, Japan; g ARSOA Research & Development Center, ARSOA Keioh Group Corporation, Hokuto, Yamanashi, Japan; Queens College CUNY

## Abstract

Lentilactobacillus kosonis NBRC 111893 is a species of heterolactic acid bacteria isolated from kôso, a Japanese sugar-vegetable fermented beverage. The draft genome sequence of *L. kosonis* NBRC 111893 is useful for understanding the features of the genus *Lentilactobacillus* and its possible uses in fermented foods.

## ANNOUNCEMENT

Lentilactobacillus kosonis NBRC 111893 is a heterolactic acid bacterial species, isolated from 7-day fermented kôso (1, 2). *L. kosonis* NBRC 111893 was cultured in pure form in MRS broth (Difco Laboratories) under aerobic conditions (unmodified atmosphere) at 30°C for 2 days for genomic DNA sequencing. The extraction process based on the work of Morita et al. (3) is detailed in our previous report (1). Whole-genome sequencing of *L. kosonis* NBRC 111893 was performed using an Ion Torrent PGM system with the Ion Xpress Plus fragment library kit (Thermo Fisher). The read length was 200 bases. The total read data were processed using the Newbler v2.8 assembler (Roche), and default parameters were used for all software unless otherwise specified (4). A total of 2,057,652 reads were assembled into 21 contigs. The average contig size was 93,891 bp, the *N*_50_ contig size was 471,655 bp, and the longest contig was 556,830 bp. The resulting draft genome sequence was 1,971,719 bp, with an average read coverage of 302.53×. The average G+C content was 37.9%. The draft genome of *L. kosonis* NBRC 111893, annotated using the RAST server (https://rast.nmpdr.org/) and Glimmer v3 with default settings, contains 2,563 candidate open reading frames and 53 tRNA genes (5).

Lentilactobacillus kosonis is a heterofermentative lactic acid bacterium that produces lactic acid and acetic acid during fermentation. Two copies of d*-*lactate dehydrogenase genes and two copies of l*-*lactate dehydrogenase genes were annotated in the genome of *L. kosonis* NBRC 111893, suggesting that it can produce both d- and l*-*lactic acid; and four copies of acetate kinase genes were found, indicting the possible production of acetic acid from acetyl phosphate. One copy of a lactate 2-monooxygenase gene was found in the *L. kosonis* genome, showing that lactic acid might be converted into acetic acid with carbon dioxide released.

Various species of the genus *Lentilactobacillus* have been isolated from silage and fermented vegetables and are reported to have a free-living lifestyle (6). *L. kosonis* NBRC 111893 closely matches the features of the genus *Lentilactobacillus* in that *L. kosonis* NBRC 111893 was isolated from a fermented beverage, and four copies of genes for agmatine deaminase were also found in the genome.

Lentilactobacillus kosonis NBRC 111893 has been annotated with 10 copies of dipeptidase (EC 3.4.13.−) genes, including a d*-*alanyl-d*-*alanine dipeptidase gene and a proline dipeptidase gene. Nine copies of aminopeptidase (EC 3.4.11.−) genes were also found in the *L. kosonis* genome, including three copies of tripeptide aminopeptidase genes, two copies of lysyl aminopeptidase genes, two copies of d-stereospecific aminopeptidase genes, one copy of a methionine aminopeptidase gene, and one copy of a proline aminopeptidase gene. Three copies of bleomycin hydrolase (aminopeptidase C) genes were also found in the *L. kosonis* genome (7). This genomic information reveals *L. kosonis* NBRC 111893 to contain several kinds of dipeptidase genes, aminopeptidase genes, and even bleomycin hydrolase genes. *L. kosonis* NBRC 111893 appears to have potential use for the fermentation of various foods (8).

### Data availability.

The genome sequences of *Lentilactobacillus kosonis* NBRC 111893 have been deposited in the DDBJ/EMBL/GenBank under accession number BEXA00000000. The version described in this paper is version BEXA01000000. The raw reads of *Lentilactobacillus kosonis* NBRC 111893 have been deposited in the Sequence Read Archive (SRA) via the DDBJ system under accession number DRR153269.
